# 179. Identification and Whole Genome Sequencing Analysis of an Oxacillinase (OXA)-48-like-producing *Acinetobacter baumannii* Outbreak in California, January-May 2021

**DOI:** 10.1093/ofid/ofab466.179

**Published:** 2021-12-04

**Authors:** Diana Holden, Matthew Sylvester, John Crandall, Fengfeng Xu, Emily C Schneider, Hillary Berman Watson, Peng Zhang, Jaclyn Bacud, Rafael Mejia, Erin Epson, Zenda Berrada, Tisha Mitsunaga, Rituparna Mukhopadhyay

**Affiliations:** 1 California Department of Public Health, Richmond, CA; 2 Washington State Department of Health, Shoreline, Washington

## Abstract

**Background:**

In January 2021, a California acute care hospital (ACH A), a sentinel site for *Acinetobacter baumannii* (AB) surveillance, identified OXA-48-like-carbapenemase producing (CP) AB in a patient admitted from a ventilator-equipped skilled nursing facility (vSNF A); OXA-48-like AB had not been previously reported in the United States.

**Methods:**

Our investigation included onsite infection control (IC) assessments, contact tracing, and point prevalence surveys (PPS) at vSNF A. The Antibiotic Resistance (AR) Laboratory Network performed carbapenemase testing on AB isolates (including those from ACH A) and PPS swabs. A case was defined as a patient with an OXA-48-like AB isolate, or an epidemiologically-linked patient with an OXA-48-like gene detected via screening. We performed whole genome sequencing (WGS) of OXA-48-like AB and other CP organisms on the Illumina MiSeq and Oxford Nanopore MinION for short and long read sequencing, respectively.

**Results:**

Since January 2021, we have identified five OXA-48-like AB cases (including the index), six OXA-48-like cases (no organism recovered), and six patients with other CP organisms at ACH A and vSNF A. Since August 2019, vSNF A has concurrently been experiencing an OXA-109 AB outbreak. A second vSNF A patient, Patient 2, who overlapped with the index patient, had OXA-48-like *Klebsiella pneumoniae* (KP) (November 2019) and OXA-109 AB (May 2020) isolates. WGS of the index patient’s AB and Patient 2’s KP isolates identified a rare OXA-48-like gene located on the AB chromosome and a KP plasmid. The OXA-48-like AB was also carrying an OXA-109 gene, and hqSNP analysis indicated it varied by 9-44 single-nucleotide polymorphisms (SNPs) from 14 OXA-109 AB isolates linked to that outbreak, and 0-3 SNPs from the other OXA-48-like AB case isolates.

Figure 1. Phylogenetic Tree Comparison of OXA-109 AB and OXA-48-like AB Isolates

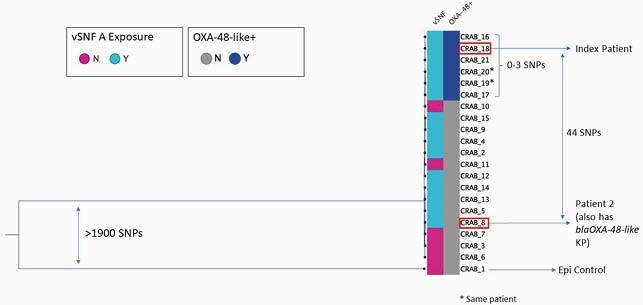

Figure 2. Epidemic Curve of OXA-109 AB, OXA-48-like AB, and Other CP Organism Cases, 2019-2021

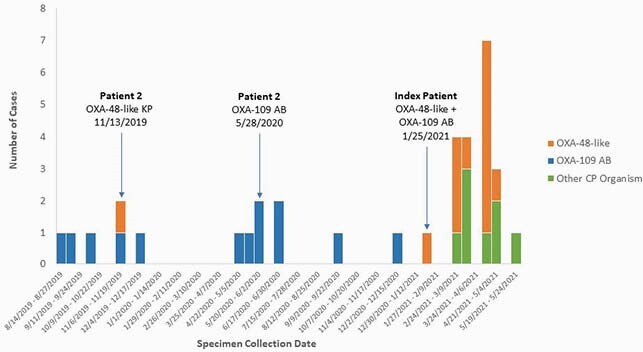

**Conclusion:**

The first reported case of OXA-48-like AB in the US was identified through public health sentinel laboratory surveillance, allowing prompt response to contain spread of a novel multidrug-resistant organism (MDRO). WGS detected a rare OXA-48-like gene in AB and KP and provides evidence for possible interspecies transfer of this gene from KP to AB through plasmid transfer followed by chromosomal integration.

**Disclosures:**

**All Authors**: No reported disclosures

